# Confirmation of translatability and functionality certifies the dual endothelin1/VEGFsp receptor (DEspR) protein

**DOI:** 10.1186/s12867-016-0066-8

**Published:** 2016-06-14

**Authors:** Victoria L. M. Herrera, Martin Steffen, Ann Marie Moran, Glaiza A. Tan, Khristine A. Pasion, Keith Rivera, Darryl J. Pappin, Nelson Ruiz-Opazo

**Affiliations:** Whitaker Cardiovascular Institute, Boston University School of Medicine, 700 Albany Street, Boston, MA 02118 USA; Department of Medicine, Boston University School of Medicine, 700 Albany Street, Boston, MA 02118 USA; Department of Pathology and Biomedical Engineering, Boston University, Boston, USA; Cold Spring Harbor Laboratory, 1 Bungtown Road, Cold Spring Harbor, NY 11724 USA

**Keywords:** DEspR, Pseudogene, DEspR protein–protein interactions

## Abstract

**Background:**

In contrast to rat and mouse databases, the NCBI gene database lists the human dual-endothelin1/VEGFsp receptor (DEspR, formerly *Dear*) as a unitary transcribed pseudogene due to a stop [TGA]-codon at codon#14 in automated DNA and RNA sequences. However, re-analysis is needed given prior single gene studies detected a tryptophan [TGG]-codon#14 by manual Sanger sequencing, demonstrated DEspR translatability and functionality, and since the demonstration of actual non-translatability through expression studies, the standard-of-excellence for pseudogene designation, has not been performed. Re-analysis must meet UNIPROT criteria for demonstration of a protein’s existence at the highest (protein) level, which a priori, would override DNA- or RNA-based deductions.

**Methods:**

To dissect the nucleotide sequence discrepancy, we performed Maxam–Gilbert sequencing and reviewed 727 RNA-seq entries. To comply with the highest level multiple UNIPROT criteria for determining DEspR’s existence, we performed various experiments using multiple anti-DEspR monoclonal antibodies (mAbs) targeting distinct DEspR epitopes with one spanning the contested tryptophan [TGG]-codon#14, assessing: (a) DEspR protein expression, (b) predicted full-length protein size, (c) sequence-predicted protein-specific properties beyond codon#14: receptor glycosylation and internalization, (d) protein-partner interactions, and (e) DEspR functionality via DEspR-inhibition effects.

**Results:**

Maxam–Gilbert sequencing and some RNA-seq entries demonstrate two guanines, hence a tryptophan [TGG]-codon#14 within a compression site spanning an error-prone compression sequence motif. Western blot analysis using anti-DEspR mAbs targeting distinct DEspR epitopes detect the identical glycosylated 17.5 kDa pull-down protein. Decrease in DEspR-protein size after PNGase-F digest demonstrates post-translational glycosylation, concordant with the consensus-glycosylation site beyond codon#14. Like other small single-transmembrane proteins, mass spectrometry analysis of anti-DEspR mAb pull-down proteins do not detect DEspR, but detect DEspR-protein interactions with proteins implicated in intracellular trafficking and cancer. FACS analyses also detect DEspR-protein in different human cancer stem-like cells (CSCs). DEspR-inhibition studies identify DEspR-roles in CSC survival and growth. Live cell imaging detects fluorescently-labeled anti-DEspR mAb targeted-receptor internalization, concordant with the single internalization-recognition sequence also located beyond codon#14.

**Conclusions:**

Data confirm translatability of DEspR, the full-length DEspR protein beyond codon#14, and elucidate DEspR-specific functionality. Along with detection of the tryptophan [TGG]-codon#14 within an error-prone compression site, cumulative data demonstrating DEspR protein existence fulfill multiple UNIPROT criteria, thus refuting its pseudogene designation.

**Electronic supplementary material:**

The online version of this article (doi:10.1186/s12867-016-0066-8) contains supplementary material, which is available to authorized users.

## Background

In contrast to rat and mouse databases listing Dear as a gene, DNA and RNA sequence databases list the human Dear gene or the dual-endothelin1/VEGFsp receptor (DEspR) as a pseudogene [1] (Additional file 1: Figure S1). Automated DNA sequence databases report a stop codon [TGA] instead of tryptophan [TGG] at codon#14 reported in single gene study [2]. The current NCBI pseudogene annotation updated in May 2016 and referenced in other sites is discrepant with the single research group single-gene studies of human DEspR showing expression in human kidney via immunohistochemistry using a polyclonal anti-DEspR antibody, and functional studies of human DEspR expressed in permanent Cos1 cell transfectants detecting the predicted protein size by Western blot analysis as well as binding to DEspR-ligands (endothelin-1 and VEGFsp) [2]. The NCBI pseudogene annotation is also discrepant with the single gene study demonstrating DEspR-specific functional roles in cancer and putative regulation at the splicing level with detection of both unspliced and spliced DEspR RNA in human tumor cells by allele-specific amplification-refractory mutation system (ARMS) methodology [3].

Experimental clarification is warranted since the basis for the NCBI pseudogene annotation, automated DNA/RNA-sequencing, is known to have reproducible systematic sequencing errors, regardless of technology [4]. Occurrences of, hence risks for, systematic errors eliminate the a priori assumption that multiple occurrences negate errors. More specifically, systematic errors in high throughput DNA sequencing has been observed to occur “even in overlapping paired reads from high-coverage data, approximately one in 1000 bp, and are highly replicable across experiments” [4]. Moreover, given discrepancies among methodologies, the Sanger sequencing is the final determinant of sequence discrepancy since “any difference from the Sanger sequence is defined as a sequencing error” [5].

Further support for the need for scientific clarification is found in the GENCODE Pseudogene Resource which states that “the definition of a pseudogene is based on the presence of specific characteristics such as premature stop codon, coding sequence frame shift, truncation, or disabling insertion/deletion—*unless evidence (transcriptional, functional, publication) shows that the locus represents a protein-coding gene*” [6]. Concordant with Pei et al. [6], Kageyama et al. [7] explicitly states that “before a particular transcript can be determined to be a long non-coding RNA (or ‘transcribed pseudogene’), there must be somewhat convincing evidence for its lack of translatability.” Hence, deductions from automated sequence databases need to be evaluated in the context of experimental evidence for translatability and functionality.

To address the need for scientific clarification of DEspR (Dear) as a gene or pseudogene, we therefore upheld established standards that (1) “*the translatability of the candidate can be validated with specific antibodies against amino acid sequences predicted from the ORF*,” [7] and that (2) “*an assessment of … a protein’s molecular activity by biochemical methods should be the final certification of an active gene product*” [7]. These perspectives are codified in the Central Protein Resource UNIPROT criteria for translatability: “evidence for existence of a protein at the protein level, such as via antibody detection, is the highest level of evidence” [8].

Here we confirm the existence of the DEspR gene product at the nucleotide and protein level. We show that both Sanger and Maxam–Gilbert sequencing detect two G’s for a tryptophan [TGG]-codon#14. We also determine that some RNA-seq entries also contain two G’s but with an extra A or TA, as the region in question spans a canonical Yamakawa compression motif [9]. Monoclonal anti-DEspR antibodies to different DEspR epitopes detect the identical glycosylated 17.5 kDa pull-down protein from membrane-bound proteins from human tumor cells and the Cos1-DEspR + permanent transfectants cells. More importantly, DEspR molecular activity, protein–protein interactions, protein-specific properties and functionality are shown, and found to play key roles in cancer stem cell anoikis resistance and growth.

## Results

### Confirmation of two G’s and compression site

**Fig. 1:**
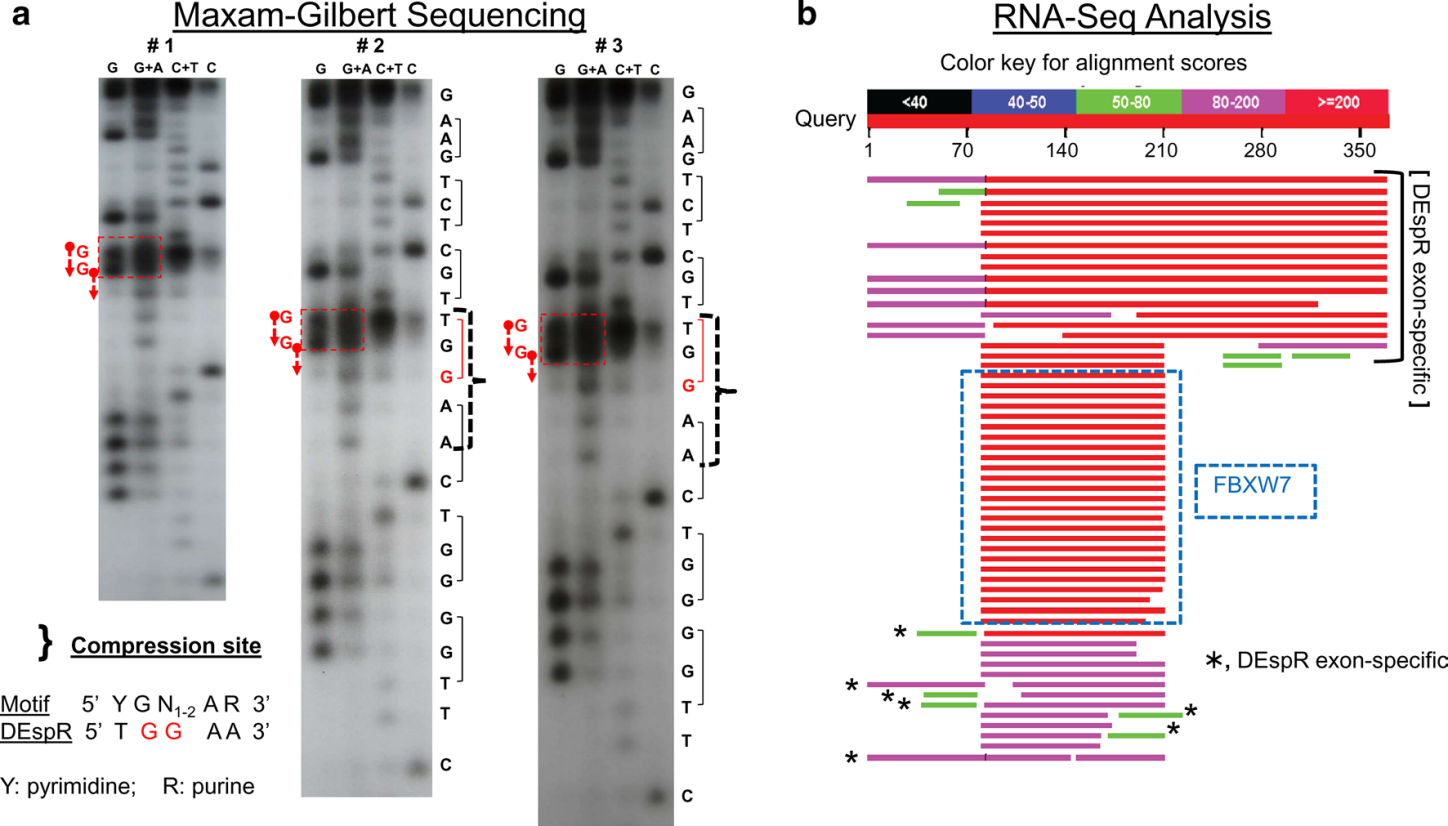
DEspR DNA and RNA sequence analysis. **a** Maxam–Gilbert DNA nucleotide sequence analysis in three different gel runs of increasing wattage (#1 = 25 watts; #2 = 35 watts; #3 = 50 watts) spanning controversial region shows: consistent area of compression (}) which contains the Yamakawa compression-motif. The compressed two G’s and single T are depicted in codon#14: TGG.], codons; the two G’s (in *red*). **b** Representative RNA-Seq analysis of 727 unedited RNA-seq entries show DEspR exon-specific RNAs distinguished from the anti-sense strand transcript, FBXW7, exon-specific sequence. Query DEspR sequence spans 1–372 nt of DEspR transcript

To determine whether the two G’s are detected forming tryptophan [TGG]-codon#14, as observed in Sanger sequencing (Additional file 2: Figure S2), we performed manual Maxam–Gilbert sequencing using 8 % denaturing polyacrylamide sequencing gels on ^32^P end-labeled PCR-amplified cDNA electrophoresed at three different fixed wattages (Fig. 1a). Clearly, the two G’s are noted with the corresponding C bands that accompany most G nucleotides. Interestingly, the compression in the gel run is consistently observed in Sanger sequencing and in Maxam–Gilbert sequencing, and contains the compression motif found in 68 % of sequencing errors with compression [9] (Fig. 1a; Additional file 2: Figure S2). This compression results in the “slippage’ of the two G’s with a T nucleotide, thus leaving a space in the gel read. Additionally, the compression region is within a 3-nt stem-loop structure that could render this region prone to sequencing errors which are not present in rat and mouse DEspR gene sequences, both of which also contain the identical tryptophan [TGG]-codon#14 (Additional file 2: Figure S2).

Importantly, queries against subsets of the NCBI sequence read archive (SRA) database reveals DEspR-specific exon sequences which would not be expected for the FBXW7 transcript in the opposite strand (Fig. 1b). Notably, five entries show the two G’s out of 124 data entries in said file (Additional file 3: Figure S3), and that these two G’s are associated with an insertion of a “T–A’ or an “A” (Additional file 3: Figure S3). These observations show that the two G’s are indeed detected and are located within a problematic sequencing region, concordant with observations in manual sequencing gels of nucleotide compression spanning a known sequence motif for compression [9]. These data show the questioned two G’s in support of tryptophan [TGG] codon in 5/124 sequences similar to manual sequencing runs. Given that Sanger-sequencing is the accepted standard final determinant of nucleotide discrepancies [5], these experimental data support the need for clarification and demonstration of DEspR protein expression.

### Analysis of translatability and protein-specific properties

**Fig. 2:**
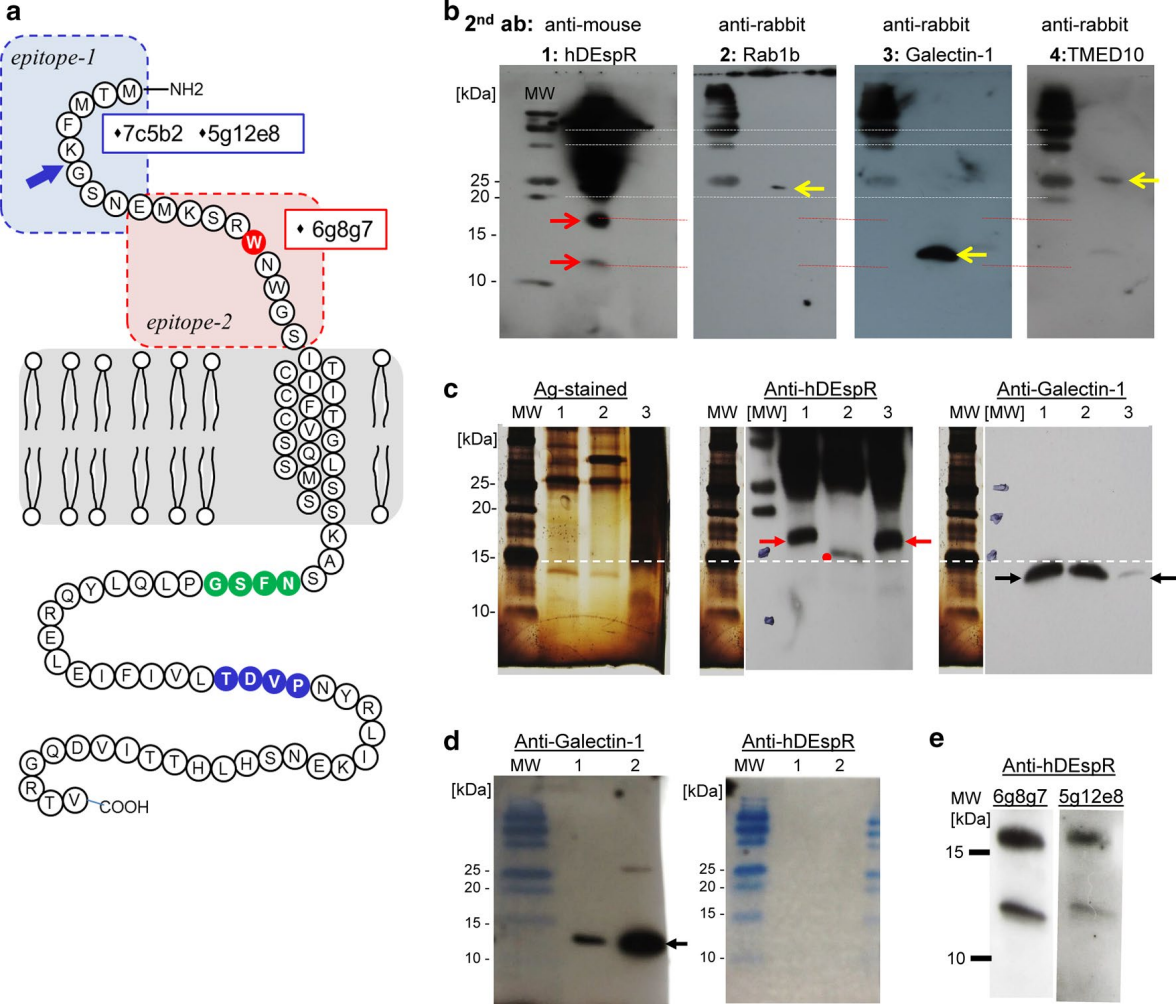
Analysis of DEspR translatability. **a** Schematic diagram of DEspR protein and mAb-epitopes. Two distinct peptides (*epitope*-*1, epitope*-*2*) in the extracellular domain were used to develop murine monoclonal antibodies (mAbs). Two high-affinity mAbs target human-specific epitope-1: 7c5b2, 5g12e8; and one high-affinity mAb targets the pan-species reactive epitope-2, identical in human, monkey, and rat. Epitope-2 spans the putative ligand binding domain [24]. The 5g12e8 mAb was used in pull-down experiments; 5g12e8 and 6g8g7 were used in Western blot analyses, 7c5b2 mAb was used in FACs analysis, immunostaining, and internalization assays, and all three were used in functional inhibition assays. The contested tryptophan (W)-aa#14 (*red*); consensus glycosylation site sequence: (*green*, N-F-S-G), known internalization recognition sequence: (*blue*: T-D-V-P). A* blue arrow* marks the splice junction between exon1 and exon2, i.e. between amino-acids G and K (aa#5-#6). **b** Sequential Western blot analyses of pull-down proteins from glioblastoma U87 membrane proteins using different antibodies specific for proteins identified by mass spectrometry analysis of pull-down protein-products. The identical blot was sequentially probed, stripped of antibody, confirmed as stripped, then re-probed in the following order: #1: anti-hDEspR-5g12e8 mouse mAb, #2: anti-Rab1b rabbit polyclonal Ab (pAb), #3: anti-Galectin-1 rabbit pAb; #4: anti-TMED10 rabbit pAb. Molecular weight markers are noted. DEspR bands are ~17.5 and 12.5 kDa. Expected sizes are detected for Rab1b: 22 kDa, Galectin-1: 14 kDa, and TMED10: 25 kDa. **c** *Panel*-1 shows silver-stained gel of pull-down protein products using 5g12e8 mAb from membrane proteins isolated from: (1) glioblastoma U87 CSCs, (2) PNGase-digested sample of pull-down proteins from U87 CSCs, (3) permanent transfectants DEspR-positive Cos1-cells. *Panel*-2 shows Western blot analysis using anti-DEspR 5g12e8 mAb showing DEspR band (*lane 1*), smaller DEspR + band after PNGase digest-samples (*lane 2*), and identically-sized DEspR band in DEspR-positive Cos1-cell permanent transfectants showing appropriate splicing and translatability of DEspR-minigene transfected into Cos-1 cells (*lane 3*). *Panel*-3 Western blot analysis of different wells in the same gel run probed with anti-Galectin-1 pAb showing distinct sized protein bands, thus confirming DEspR-specific bands are not Galectin-1 protein bands, and that Galectin-1 is not glycosylated as reported. **d** Western blot analysis of Galectin-1 recombinant protein. *Panel 1* overlay of gel-image and western blot image showing detection of Galectin-1 recombinant protein at expected size 14 kDa. *Panel 2* overlay of gel-image and western blot image probed with anti-DEspR 5g12e8 mAb showing non-cross reactivity of anti-hDEspR mAb with Galectin-1. **e** Sequential western blot analysis of 5g12e8-pull-down proteins from U87 CSCs probed first with 6g8g7 (*left panel*), and subsequently with 5g12e8 after ‘stripping’ (*right panel*), detects identical protein bands. This confirms that 6g8g7 epitope is on the same protein as 5g12e8 epitope, thus corroborating DEspR protein existence

In order to clarify the existence of DEspR protein complying with established UNIPROT criteria for detection of protein by antibody made from deduced amino acid sequences, we performed anti-DEspR mAb pull-down and subsequent Western blot and mass spectrometry analyses of pull-down products from cell membranes. Membranes were isolated from human glioblastoma tumor cells and U87 cancer stem-like cells (CSC) which we isolated and characterized for CSC tumor initiating properties [3]. We performed pull-down experiments using an anti-human DEspR specific antibody, 5g12e8 mAb (Fig. 2a), which detected DEspR on prior Western blot analysis of CSC membrane proteins [3].

Analysis of pull-down products by mass spectrometry (MS) (Table 1; Additional file 4: Table S1) revealed that the DEspR-protein interacts with several proteins involved in intracellular trafficking, angiogenesis, and/or cancer: vimentin, Gal-3, Gal-1 and TMED10. Although MS analyses did not detect DEspR (Table 1), Western blot analyses of pull-down products detected DEspR protein bands at ~17.5 and ~12.5 kDa, larger than the deduced amino acid sequence predicting ~10 kDa, and distinct from the other proteins in the pull-down-complex: Rab-1b (22 kDa), Galectin-1 (14 kDa) and TMED10 (25 kDa) (Fig. 2b). Data indicate that DEspR is likely glycosylated given its consensus glycosylation site [N-F-S-G] (Fig. 2a), and clearly distinct from the other proteins in the pull-down complex as evidenced by size, relative abundance, and the lack of antibody cross-reactivity (Fig. 2b). Glycosylation of DEspR is confirmed after peptide-N-glycosidase F (PNGase F) treatment of pull-down proteins showing decrease in size of DEspR upon Western blot analysis (Fig. 2c, middle panel, lane 2). These data validate the canonical glycosylation site beyond the purported pseudogene stop codon#14, and provides a mechanism for DEspR-Galectin-1 and DEspR-Galectin-3 binding as observed in the pull-down products.

**Table 1:** Proteins pulled-down with anti-DEspR mAb 5g12e8 from U87 CSC membranes

Accession	%	#SM	#P	#AAs	Mass	Score	Description
P08670	55.8	36	31	466	53,619	177.59	*Vimentin (VIM)*
P60709	34.4	11	9	375	41,709	66.79	*Actin, cytoplasmic 1 (ACTB)*
P68371	41.6	13	12	445	49,799	56.53	Tubulin beta-4B chain (TUBB4B)
P07437	35.1	11	10	444	49,638	47.84	*Tubulin beta chain (TUBB)*
Q13885	29.0	10	9	445	49,874	42.11	Tubulin beta-2A chain (TUBB2A)
P17931	25.6	7	5	250	26,136	39.26	Galectin-3 (LGALS3)
P09382	49.6	7	6	135	14,706	38.90	*Galectin-1 (LGALS1)* ^a^
Q13509	25.6	8	8	450	50,400	32.86	Tubulin beta-3 chain (TUBB3)
Q9BUF5	15.7	6	5	446	49,825	21.46	Tubulin beta-6 chain (TUBB6)
P61006	13.0	2	2	207	23,653	12.01	Ras-related protein Rab-8A (RAB8A)
C9J8S3	15.0	2	2	160	18,015	11.01	*Ras-related protein Rab-7a (RAB7A)*
P61026	6.0	1	1	200	22,526	9.59	*Ras-related protein Rab-10 (RAB10)*
P51153	5.9	1	1	203	22,759	7.60	Ras-related protein Rab-13 (RAB13)
H0YNE9	6.4	1	1	188	21,854	7.51	Ras-related protein Rab-8B (Fragment) (RAB8B)
G3V2K7	7.2	1	1	153	16,893	5.96	*Transmembrane emp24 domain-containing protein 10 (TMED10)* ^a^
Q9Y3B3-2	5.3	1	1	188	21,219	4.85	Transmembrane emp24 domain-containing protein 7 (TMED7)
Q9BVK6	3.8	1	1	235	27,260	4.36	Transmembrane emp24 domain-containing protein 9 (TMED9)
F8WBC0	34.4	1	1	32	3501	2.90	*Ras-related protein Rap-1b (Fragment) (RAP1B)*

Proteins in italic face letters are proteins detected in three independent pull-down experiments

*%* the percentage of the protein sequence covered by identified peptides; *#SM* the number of peptide spectrum matches; *#P* the total number of distinct peptide sequences identified in the protein group; *#AAs* number of amino acids; Mass, mass in Daltons; Score, the sum of the ion scores of all peptides that were identified

^a^Proteins verified by Western blot analysis of pull-down proteins; Rab-1b was detected in two independent experiments and verified by Western blot analysis (Fig. 2b)

However, because of the abundance of Galectin-1 in the pull-down products, we performed more Western blot analyses to eliminate the possibility that anti-DEspR antibody cross reacts with Galectin-1 and vice versa. As shown in Fig. 2c, the anti-human DEspR 5g12e8 mAb detects the 17.5 kDa glycosylated DEspR in the pull-down products from U87-CSC cell membrane proteins and from the membrane proteins isolated from DEspR-positive Cos1-permanent transfectant cells. These DEspR + Cos1-permanent transfectants cells were previously shown to express human DEspR by immunostaining, and to competitively bind ligands and anti-DEspR (7c5b2) mAb [2]. After PNGase F digestion, 5g12e8 also detected the smaller, hence deglycosylated DEspR (Fig. 2c, middle panel). In contrast, anti-Galectin-1 mAb detected a 14 kDa protein band with markedly different size and expression levels to DEspR in all three lanes (Fig. 2c, right panel).

To further confirm this distinction, we performed double Western blot analyses of Galectin-1 recombinant protein. As shown in Fig. 2d, anti-Galectin-1 mAb detected human Galectin-1, in contrast to the anti-human DEspR 5g12e8 mAb which did not cross react to both 100 and 500 ng of purified recombinant human Galectin-1 on Western blot analysis, thus confirming that DEspR in the pull-down product is distinct from other pull-down products (Fig. 2b) especially Galectin-1 (Fig. 2c). Additionally, since Galectin-1 is known not to be glycosylated [10] as confirmed in Fig. 2c, the PNGase F-treated sample showing a decrease in size cannot, therefore, be Galectin-1.

To further corroborate the detection of human DEspR protein in human tumor cells, we performed sequential probing of the identical Western blot with two different anti-human DEspR mAbs targeting different epitopes. As shown in Fig. 2e, the 6g8g7 mAb targeting the epitope spanning the disputed tryptophan [TGG]-codon#14 (Fig. 2a) detects both the glycosylated 17.5 kDa and the less glycosylated 12.5 kDa DEspR bands. After removal of the 6g8g7 antibody and demonstration of no residual signals, the Western blot was re-probed with the 5g12e8 mAb used in the pull-down experiment and which targets a human DEspR-specific epitope spanning a peptide present only if correctly spliced (Fig. 2a). This 5g12e8 probed western blot detected the identical protein bands (Fig. 2e), thus showing that two different anti-hDEspR mAbs 6g8g7 and 5g12e8, which target different epitopes, detect the same protein bands representing spliced and glycosylated DEspR.

### Protein–protein interactions: analysis of co-localization of DEspR and Galectin-1

**Fig. 3:**
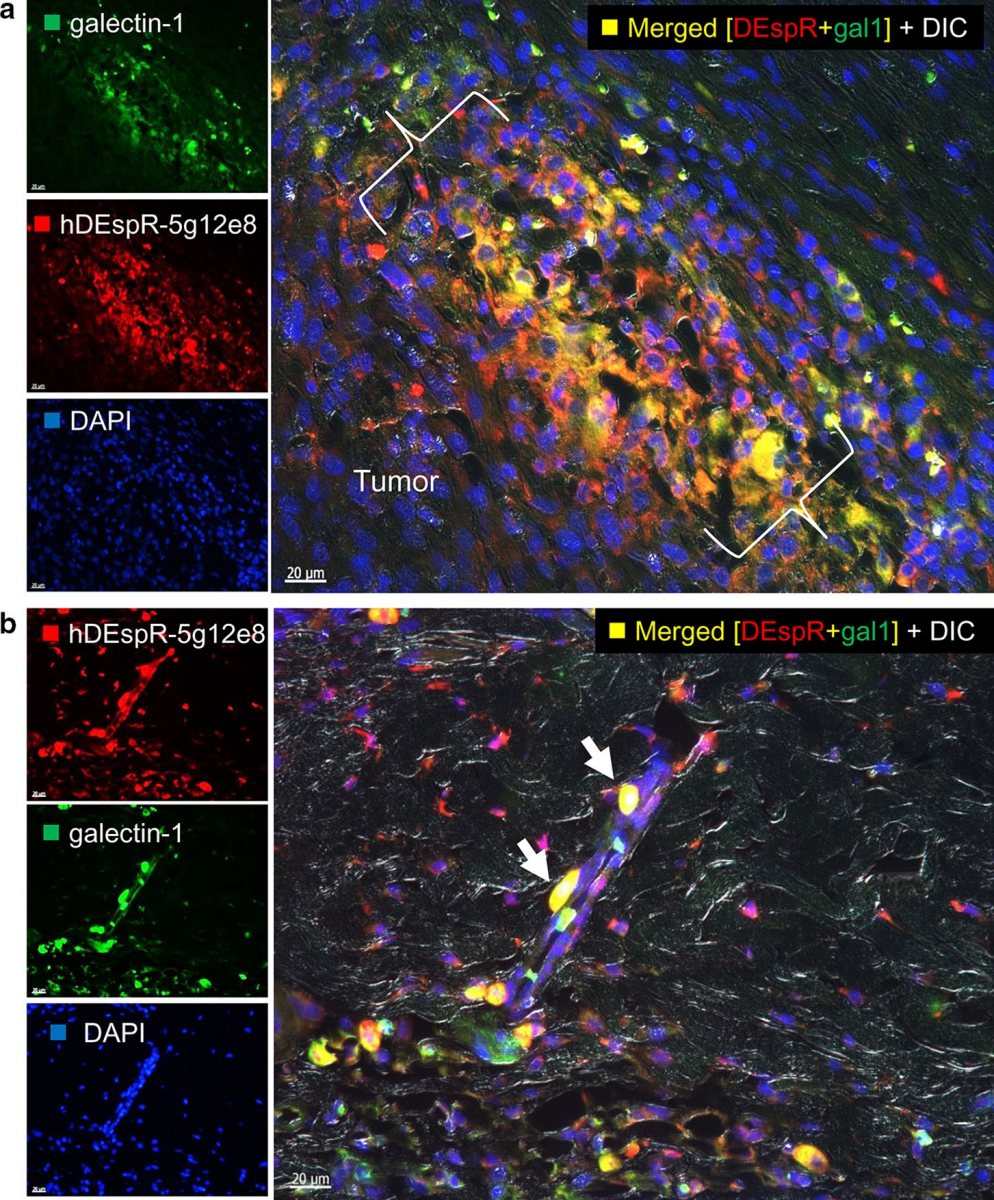
Dual-fluorescence co-immunostaining analysis of DEspR and Galectin-1 expression. **a** Analysis of the expanding tumor zone of a U87-CSC xenograft tumor invading through the tumor fibrous cap. Co-localization (*yellow*, *yellow dotted circle*) of increased human-DEspR expression (*red dotted circle*) in invasive U87 tumor cells and Galectin-1 (*green dotted circle*) is observed in the invasive front. {}, invasive tumor front. Host subcutaneous tissue is to the* upper right* corner. **b** Co-localization of hDEspR and Galectin-1 is detected in tumor cells adhering to the outer wall of a microvessel in the subcutaneous tissue demonstrating invasive nature of U87 in xenograft tumors and homing to microvessels

Since both DEspR and Galectin-1 are “pulled-down” consistently (n = 6), we next determined whether they are co-localized in tumors. We performed double immunostaining of xenograft tumors derived from U87-CSCs [3] using human specific anti-human DEspR mAb 7c5b2 and human-specific anti-Galectin-1 antibody. As shown in Fig. 3a, DEspR and Galectin-1 are indeed co-localized in tumors, and located more in the expanding tumor zone. Additionally, as shown in Fig. 3b, DEspR and Galectin-1 are co-expressed in tumor cells that have invaded through the xenograft tumor cap and adhered onto a microvessel in the surrounding subcutaneous tissue of the host nude rat. The detection of co-localization is concordant with the pull-down of both DEspR and Galectin-1 in multiple independent experiments (Table 1).

### Analysis of functionality at multiple levels certifies DEspR protein

**Fig. 4:**
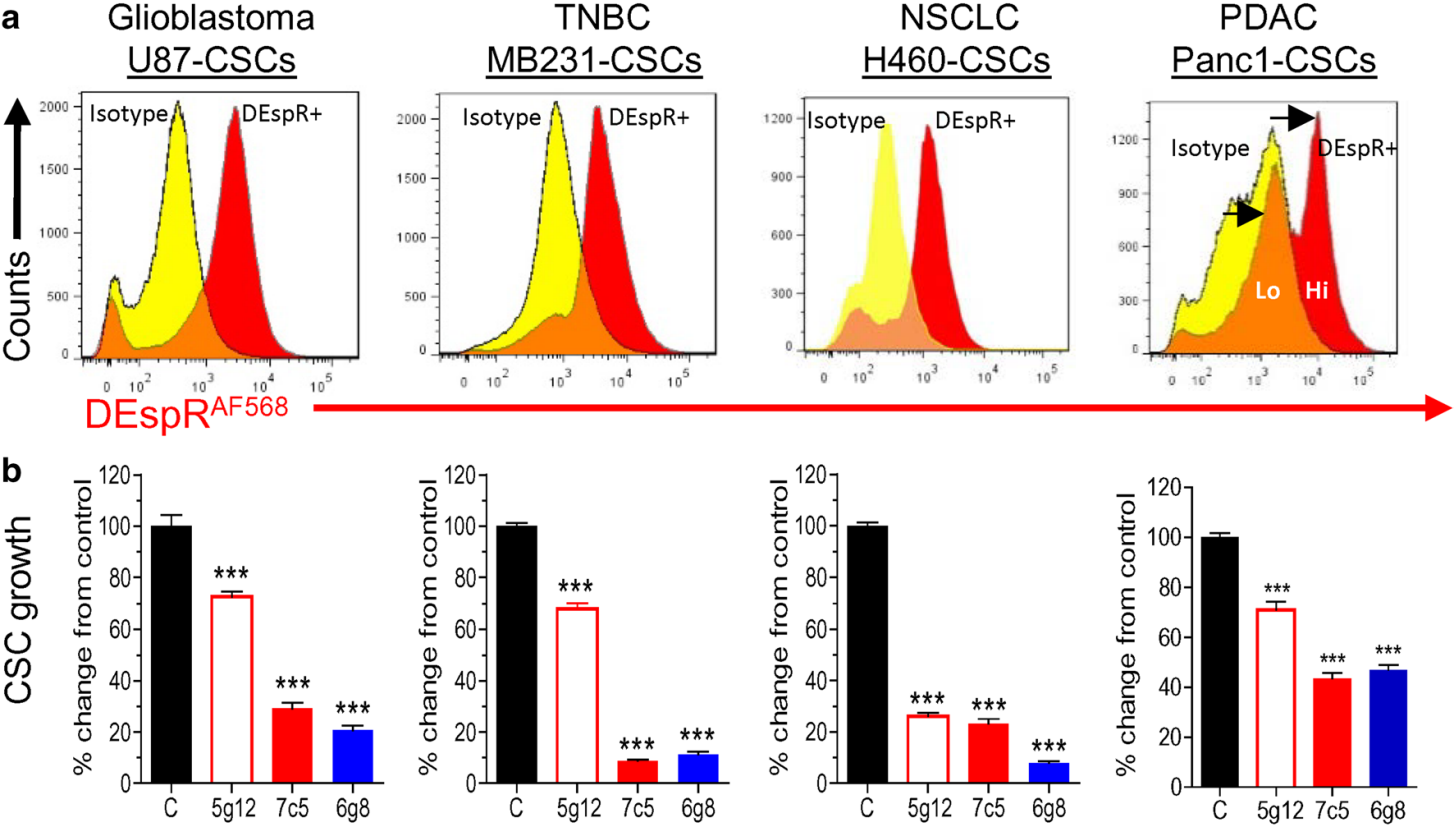
Demonstration of DEspR protein and functionality in different human tumor cell lines by anti-DEspR mAbs. **a** FACs analysis of different cancer tissue type CSCs: glioblastoma, triple negative breast cancer (TNBC), non-small cell lung cancer (NSCLC), and pancreatic ductal adenocarcinoma (PDAC) using AF-568 labeled murine anti-DEspR mAbs compared with AF568-IgG2b murine isotype control. In Panc1 CSCs, low and high DEspR + CSCs are detected. **b** Multiple murine anti-DEspR mAbs (5g12e8, 7c5b2, 6g8g7) inhibit CSC growth in suspension culture determined by the number of live CSCs after 5 days of incubation with murine anti-DEspR mAbs, compared with corresponding control non-treated CSCs. Comparative analysis is presented using % change from respective controls; ***, One Way ANOVA with Tukey’s multiple comparisons test *P* < 0.0001. Epitope 1 murine mAbs: 5g12, 5g12e8, 7c5, 7c5b2; Epitope 2 murine mAbs: 6g8, 6g8e8

While we showed DEspR functionality in human tumor cells by showing that DEspR inhibition via the 7c5b2 mAb inhibited angiogenesis, tumor growth and invasiveness [3], the NCBI pseudogene designation persists (updated in May 2016). In addition to manual nucleotide DNA sequencing (Fig. 1a; Additional file 2: Figure S2), review of RNA-Seq sequence entries (Fig. 1b; Additional file 3: Figure S3), and detection of DEspR translatability or expression (Figs. 2, 3), to provide further evidence against the pseudogene designation, we further studied DEspR functionality since demonstration of functionality of a protein certifies that protein’s existence [7]. To ascertain functionality, we used different anti-human DEspR mAbs whose epitopes are depicted in Fig. 2a: 7c5b2, 5g12e8 and 6g8g7 (Fig. 4), and tested for DEspR in different cancer tissue types.

As shown in Fig. 4, we studied DEspR-inhibition effects on anoikis resistant cancer stem-like cells (CSCs) isolated from human U87 glioblastoma tumor cell line [3], human MDA-MB-231 triple negative breast cancer cell line, human H460 non-small cell lung cancer cell line and human Panc1 pancreatic ductal adenocarcinoma cell line [3]. To first demonstrate that DEspR protein is expressed on the different CSCs, we performed FACS analysis using AF568-fluorescently labeled 7c5b2 anti-DEspR mAb (Fig. 2a). Figure 4a shows that, compared to isotype control, fluorescently labeled 7c5b2-mAb specifically detected DEspR on the cell membrane in ~50 % of U87-CSCs, ~60 % of MB-231-CSCs, ~71 % of H460-CSCs and ≥55 % of Panc1-CSCs in the suspension culture conditions used.

To demonstrate the functionality of DEspR proteins present on the cell membrane, in vitro inhibition studies were performed using the 7c5b2, 5g12e8 and 6g8g7 anti-human DEspR mAbs which target different DEspR epitopes (Fig. 2a). As shown in Fig. 4b, all three mAbs significantly inhibited CSC growth in the four cancer tissue type-CSCs tested (P < 0.0001, One Way ANOVA with Tukey’s multiple comparisons test), in contrast to non-treated contemporaneous controls respectively. We note that CSC growth is representative of survival in suspension culture, proliferation and ability to form stem-cell like spheroids in suspension culture conditions. We also note some variations in the inhibition of CSC growth in vitro by the different mAbs, with 7c5b2 an 6g8g7 performing better than 5g12e8 (Fig. 4), but that all mAbs significantly inhibit survival, proliferation, and spheroid formation (CSC-growth) of U87-CSCs, MDA-MB-231-CSCs, H460-CSCs and Panc1-CSCs.

**Fig. 5:**
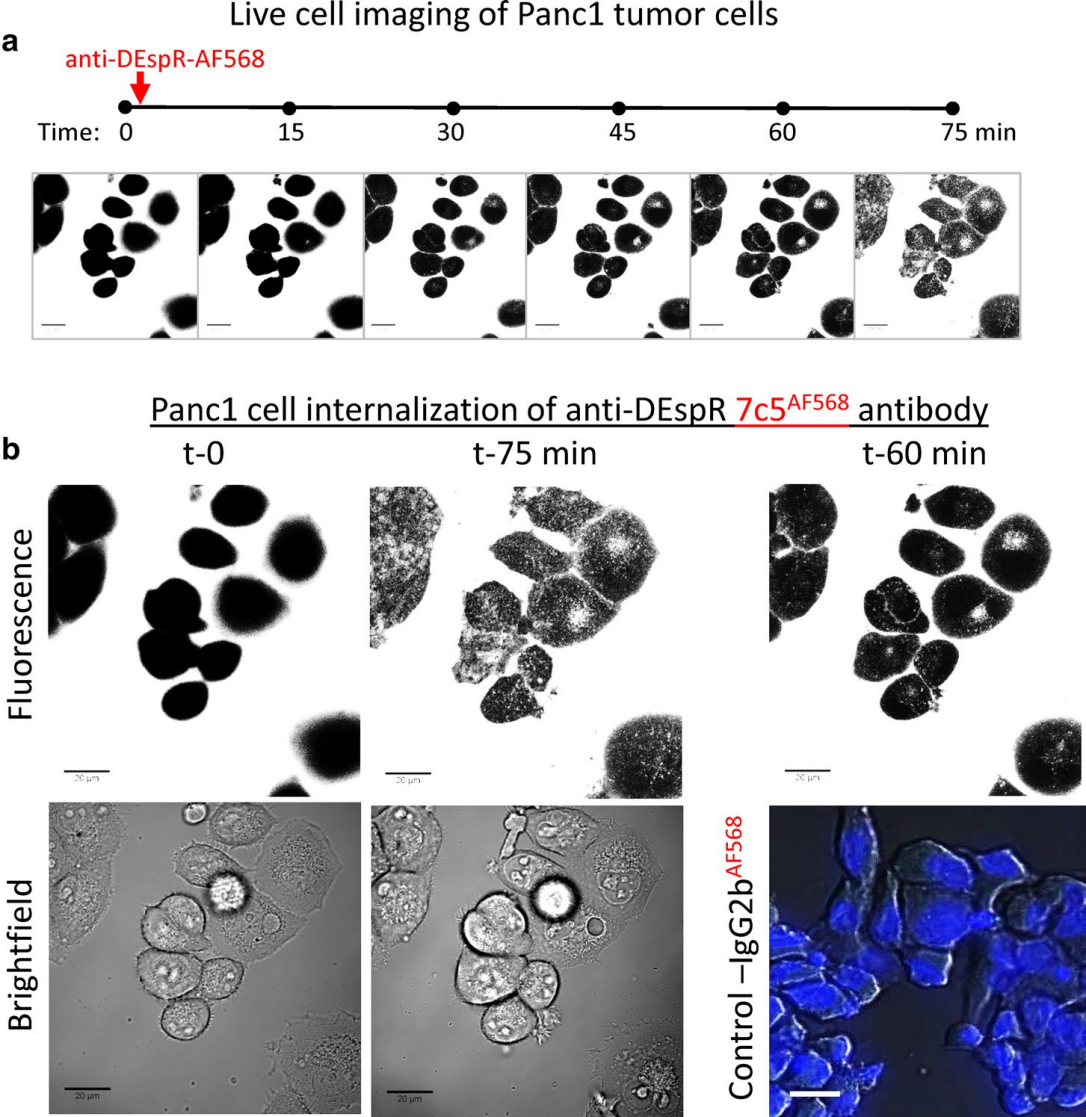
Representative time series of internalization of fluorescently labeled (AF568) anti-DEspR 7c5b2-mAb by Panc1 tumor cells within 1.5 h. **a** Confocal images showing representative Panc1 tumor cells from baseline (t-0) prior to addition of AF568-labeled antibody, up to 1 h, 15 min from addition of AF568-7c5b2 mAb. Increasing intracellular fluorescence (*white*) is detected in multiple Panc1 cells. **b** Higher magnification of Panc1 tumor cells at baseline and t-75 min with corresponding bright field images of Panc1 cells. At t-60 min, representative image of Panc1 tumor cells exposed to control AF568-labeled IgG2b isotype, with DAPI stained nuclei (*blue*) to mark cells, demonstrate no intracellular AF568 fluorescence (*red*) uptake.* Bar* = 20 microns

To further demonstrate DEspR functionality, we performed live-cell imaging of Panc1 cells using AF568-fluorescently labeled 7c5b2 mAb (Fig. 5) given the single consensus internalization-recognition sequence [T-D-V-P] in the deduced DEspR amino acid protein located beyond the contested stop codon *vs* amino acid #14-W (Fig. 2a). Demonstration of internalization would indicate translation and expression of the DEspR protein beyond the purported [TGA] stop codon#14, thus refuting the presence of the stop codon. As shown in Fig. 5a, time series of live-cell imaging shows increasing amounts of intracellular internalization beginning around 15–30 min and incrementally increasing up to 75 min after addition of fluorescent 7c5b2 anti-DEspR mAb. Higher magnification confirms intracellular fluorescence accumulation with fluorescently labeled 7c5b2 but not with isotype control IgG2b-AF568 (Fig. 5b), thus indicating AF568-labeled 7c5b2-DEspR-mediated internalization rather than non-specific endocytosis. Demonstration of internalization affirms DEspR protein functionality, and further refutes the presence of the stop codon.

## Discussion

### Stop [TGA] codon *vs* tryptophan [TGG] codon within a sequence-compression region

The detection of the two G’s to make tryptophan [TGG]-codon#14 in manual sequencing Sanger-dideoxy sequencing and Maxam and Gilbert sequencing, along with confirmation of sequence compression at the site within a Yamakawa compression-motif whether sequencing the sense strand (Maxam–Gilbert) or the antisense strand (Sanger dideoxy sequence), strongly suggests that the NCBI designation of human Dear/DEspR as a pseudogene based on stop codon#14 is erroneous. Following the accepted standard that manual Sanger sequencing defines the correct sequence rather than the automated sequence, the single gene analysis data indicates that the TGG codon is present rather than the TGA stop codon reported in automated DNA sequencing databases. This interpretation is supported by the fact that systematic sequencing errors are documented to occur with both position- and sequence-specificity even in “next generation sequencing technologies at rates greater than prior technologies” [4]. Existence of the tryptophan[TGG]-codon#14 is independently supported by detection of the DEspR protein by mAbs 5g12e8 and 7c5b2 whose peptide epitopes exist only if the DEspR RNA is spliced, and in the case of 6g8g7, only if the contested tryptophan[TGG]-codon#14 is present (Fig. 2a). Notably, 5g12e8 and 6g8g7 detect DEspR-protein as a glycosylated 12.5–17.5 kDa protein band, much greater than the 1.5 kDa predicted from a 13 amino acid peptide should there be a stop codon at codon#14. Furthermore, functional validation of the glycosylation and internalization recognition sequences beyond codon#14 (Figs. 2, 5) experimentally refute a stop codon at codon#14.

### Insights regarding other high throughput database reports

The non-detection of DEspR sequences in normal human tissue RNA-Seq databases is unsurprising since DEspR is not expressed or minimally expressed in normal tissues. Moreover, detection of unspliced DEspR-specific RNA as the dominant species [3] does not indicate non-translation, since regulation at the splicing level has recently been described for granulocyte differentiation whereby the dominant RNA species in the cell is the unspliced form [11, 12], similar to observations for detection of both spliced and unspliced DEspR-transcripts by ARMS-PCR [3].

Moreover, since DEspR overlaps with a larger transcript, FBXW7, on the opposite strand [3], RNA-seq databases that specifically exclude double stranded DNA [13] will also exclude RNA–RNA sequences such as DEspR-FBXW7 hybrids. The process-exclusion of RNA–RNA hybrids in RNA-Seq entries in current methodologies is due to the standard of excellence requiring elimination of all double stranded DNA in RNA-Seq libraries [13], which inadvertently will also exclude RNA–RNA hybrids. More specifically, since RNA–RNA hybrids can form in standard RNA isolation methods with phenol extraction [14], and can form during RNA-seq library preparation using primer annealing reactions at temperatures lower than the melting temperatures (Tm) of RNA–RNA hybrids, and given process-exclusion of any dsDNA or RNA–RNA or dsDNA-RNA hybrids, there is an inadvertent bias introduced against the detection of DEspR-specific transcripts due to RNA–RNA hybrids formed between DEspR transcript and exon#5 (or #6 depending on dataset numbering) of FBXW7 transcript on the opposite strand.

In parallel, the non-detection in proteomic databases is concordant with difficulties in detecting membrane proteins with single transmembrane domains that are less than 150 kDa [15], especially if glycosylated, since PNGase F treatments do not necessarily ensure detection by MS [16]. The current non-detection of DEspR transcripts in transcriptomics could also be due to the exclusion of RNA–RNA hybrids, and in primer pair-specific amplification due to the absence of DEspR-specific primers given the non-recognition of the DEspR gene by NCBI.

### Confirmation of translatability

Demonstration of translatability through different experimental designs and using anti-human-DEspR mAbs specific for different epitopes substantially fulfill UNIPROT criteria for determining a protein’s existence at the highest level such as detection of protein by antibody, demonstration of protein–protein interactions, demonstration of post-translational modifications, and demonstration of protein functionality [8].

Pull-down protein analysis experiments confirm the existence of DEspR via direct detection of the protein in human tumor cells by Western blot analysis of DEspR using multi-epitope anti-DEspR mAbs. The detection of the identical protein band by Western blot ‘walking’ with distinct anti-DEspR mAbs targeting two different epitopes, 5g12e8 mAb binding to DEspR epitope-1 that spans a splice junction, and 6g8g7 binding to DEspR epitope 2 that spans tryptophan[TGG]-codon#14, demonstrate the existence of the DEspR protein. If the stop codon were indeed present at codon#14 instead of tryptophan, there would simply be a 13 amino acid peptide with 1.5 kDa MW rather than the glycosylated 17.5 and 12.5 kDa protein bands, or the non-glycosylated DEspR-sequence predicted ~10 kDa protein.

Moreover, if there were indeed a stop codon at codon#14, DEspR would not be glycosylated nor internalized as the consensus sequence motifs for these sites are beyond the reported stop codon#14. The detection of the identical-sized protein on Western blot analysis of DEspR-positive Cos1 cell permanent transfectants and U87 tumor cells corroborates the protein product of the transfected plasmid DEspR mini-gene construct previously reported, and corroborates splicing of the unspliced DEspR cDNA mini-gene construct [2].

### Detection of DEspR by Western blotting but not by mass spectrometry analysis

We note that although DEspR was detected consistently in denatured conditions via Western blot analysis of membrane protein pull-down products using 5g12e8 and 6g8g7 anti-DEspR mAbs, MS did not detect DEspR-specific peptides. However, non-detection by MS does not negate DEspR protein existence for several reasons. First, based on deduced amino acid sequence predicting a single transmembrane integral membrane ~10 kDa protein that is glycosylated to 17.5/12.5 kDa, non-detection by peptide mass fingerprinting methods using techniques such as MALDI-TOF MS analysis is not surprising given that only 204 integral membrane proteins were detected on mass spectrometry in a study of rat endothelial cells at the National Center for Proteomics Research, and that no VEGF receptor-2 (151 kDa) was detected in this study [17]. Using 6718 as total number of membrane proteins [18], Mirza et al. [17] detected 3 % integral membrane proteins on MS, while Peng et al. [19] detected 301 integral membrane proteins via SDS-PAGE shotgun proteomics or 4.5 % of 6718 total membrane proteins, and highest count using SCX-RPLC-MS/MS (MudPIT) strategy detected 876 integral membrane proteins or 13 % of 6718 integral membrane proteins in murine NK cells [20]. Notably, of the ones detected by MS using an improved method that detects more membrane proteins using a centrifugal proteomic reactor, *all the proteins detected were* *>150* *kDa and* *≥2 transmembrane domains* [15].

Other factors could also account for non-detection by MS with current methodologies. As stated by Bensalem et al. [21], peptide mass fingerprinting of membrane proteins, using techniques such as MALDI-TOF MS, remains a significant challenge for at least three reasons: (1) membrane proteins are naturally present at low levels, (2) many detergents strongly inhibit proteases and have deleterious effects on MALDI spectra, and (3) despite the presence of detergent, membrane proteins are unstable and often aggregate [21]. Additionally, glycosylation of DEspR also impedes current proteomic detection as reported by Cao et al. [16] stating that “deglycosylation of plasma membrane proteins by treatment with PNGase-F did not yield detection of additional hydrophobic proteins”. Therefore, peptide mass fingerprinting MS analysis of PNGase-treated pull-down proteins which did not identify DEspR does not negate the existence of DEspR protein. These observations by others account for the non-detection by MS of the single transmembrane receptor <150 kDa DEspR protein, thus excluding MS non-detection as counter evidence for the existence of DEspR protein, especially given multiple other experimental evidence that fulfill UNIPROT criteria for determining a protein [8].

### Demonstration of protein interactions, protein-specific properties and functionality

Aside from ligand-specific activated DEspR signaling [3], detection of DEspR-protein interactions in the DEspR-Galectin-1 complex, along with other pull-down protein partners, complies with a UNIPROT criterion for ascertaining existence of a protein as a gene product through the demonstration of protein-interactions [8]. The distinct functionality of DEspR vs Galectin-1 in terms of anoikis resistance indicates DEspR protein-specific functions, rather than the potential counter-argument that DEspR functionality is merely due to the known Galectin-1 functions.

More specifically, DEspR-specific inhibition by blocking antibodies induces anoikis in four cancer tissue type CSCs, corroborating earlier observations for Panc1 and U87 CSCs [3]. In contrast, Galectin-1 is pro-anoikis [22]. Given these two observations, we hypothesize that glycosylated DEspR plays a role in anoikis resistance by binding to Galectin-1 and inhibiting its pro-anoikis function, much like glycosylation of p16-INK4a de-induces Galectin-1 pro-anoikis functions through upregulation of Galectin-3 [22]. Confirmation of the DEspR-Galectin-1 complex is demonstrated by co-localization on double-immunostaining experiments, thus further strengthening evidence for translatability and functionality by the detection of protein–protein interactions following UNIPROT criteria [8]. Distinguishing the DEspR protein band by size and quantity from its pull-down partners identified from MS by their corresponding antibodies in serial sequential probing of the identical western blot, corroborates their detection by MS, as well as eliminates the potential of erroneous cross-reactivity pull-down results.

More importantly, given that demonstration of protein functionality certifies a protein’s existence [7, 8], the demonstration of DEspR expression in >50 % of CSCs in four different cancer tissue types by FACS analysis and DEspR functionality via blocking antibody inhibition of CSC growth in all four different cancer tissue type CSCs—glioblastoma, triple negative breast cancer, non-small cell lung cancer and pancreatic ductal adenocarcinoma, provide compelling evidence of the existence of DEspR protein based on UNI-PROT criteria and standards in the field [7, 8].

Furthermore, the demonstration of internalization of DEspR protein via live cell imaging is consistent with a consensus internalization recognition sequence downstream to amino acid#14. This not only confirms translatability of the DEspR protein beyond 13 amino acids, thus refuting the stop codon presence, but also demonstrates a protein-specific property, i.e. receptor internalization and expands DEspR’s multi-functionality. Concordantly, these new data on functionality are supported by previous findings of DEspR roles in tumor angiogenesis, invasiveness, growth, as well as CSC survival in adverse conditions [3].

## Conclusions

Altogether, in the context of an error prone site at codon#14, multiple protein assays independently and collectively demonstrate the existence of the DEspR protein, and analyses that show DEspR protein properties (protein–protein interactions, glycosylation, internalization) and functional roles in multiple cancer tissue type CSCs, collectively certify the DEspR protein based on multiple UNIPROT criteria and demonstrate that the human DEspR gene is not a pseudogene. Given that fulfillment of a single UNIPROT criterion for a protein’s existence is sufficient, fulfillment of multiple criteria present compelling experimental evidence, at the highest protein level, that functional human DEspR protein exists.

## Methods

### Maxam–Gilbert sequencing

We performed Maxam–Gilbert sequencing essentially as described [23]. The hDEspR cDNA was 5′-end labelled with polynucleotide kinase using gamma-^32^P ATP. Chemical treatments for Guanine (G), Guanine + Adenine (G + A), Cytosine + Thymine (C + T) and Cytosine (C) cleavages were done as described [23]. Cleavage products from the four reactions were size separated by 8 % denaturing polyacrylamide gel electrophoresis and fragments visualized by autoradiography. Electrophoresis was performed at three different fixed wattages: 25 watts (#1), 35 watts (#2) and 50 watts (#3).

### Cell lines and antibody development and characterization by ELISA

Verified glioblastoma U87 MG (cat# ATCC HTB-14), triple negative breast cancer MDA-MB-231 (cat# ATCC HTB-26), non-small cell lung cancer NCI-H460 (cat# ATCC HTB-177) and pancreatic cancer Panc-1 (cat# ATCC CRL-1469) cell lines were obtained from ATCC. Isolation and propagation of U87, MB-231, H460 and Panc1 cancer stem cell-like cells (CSCs) was done as described [3]. CSCs were maintained and expanded through passage-5 in complete MammoCult^®^ medium (Stem Cell Technologies, BC, Canada) containing 0.5 % Methylcellulose (Stem Cell Technologies, BC, Canada) in 100 mm ultra-low attachment plates in 5 % CO2 humidified incubator at 37 °C. Testing for increased tumorigenicity was performed in vivo at passage-5. Monoclonal antibody development was custom performed by ProMab Biotechnologies (Richmond, CA). For 7c5b2 [3] and 5g12e8 mAbs we used a nine amino-acid peptide, M_1_TMFKGSN E_9_ at the amino-terminal end of hDEspR [2] and a ten amino-acid peptide, E_9_MKSRWNWGS_18_, for 6g8g7 mAb as antigens respectively. Screening of hybridoma supernatants and characterization of monoclonal antibodies were performed by ELISA using corresponding antigenic peptides. Serial dilutions of primary antibodies were incubated at 37 °C for 1 h. The wells were then incubated with horse radish peroxidase (HRP)-labeled anti-IgG (Sigma) at 37 °C for 1 h. Reactions were analyzed at 450 nm after addition of 3,3′5,5′-tetramethylbenzidine substrate at 37 °C for 10 min.

### Magnetic bead immunoprecipitation (pull-down) of DEspR protein-complex

U87 CSC membranes, Cos1-hDEspR permanent cell transfectant membranes and U87 xenograft tumor (in nude rats) membranes were isolated by differential centrifugation as described [2]. Antibody coupling to magnetic Dynabeads M-450 Epoxy (Invitrogen) was performed as per manufacturer’s instructions using 200 μg of anti-hDEspR 5g12e8 mAb per 1 mL of beads (4 × 10^8^ beads). For target binding 100 μL of 5g12e8-coupled beads (4 × 10^7^ beads) and 1.3 mg membrane protein were incubated for 2 h at 4 °C in 1 ml ice-cold PBS containing 2 mM EDTA. To capture mAb-bound protein, the tube was placed in a magnet for 2 min, the supernatant discarded and the beads washed five times with 1 mL PBS buffer containing 2 mM EDTA and 0.2 % Tween-20 at room temperature. This washed magnetic bead-‘pull-down’-protein complex was then analyzed using different methods. (a) For SDS-PAGE (18 % polyacrylamide) the beads were resuspended in 20 µL of 1X Laemmle sample buffer and proteins denatured at 65 °C × 30 min in order to size-separate and assess size(s) of protein(s) ‘pulled-down’. (b) For PNGase F (SIGMA) treatment of pull-down proteins to assess glycosylation states of proteins pulled-down, the beads were resuspended in 20 μL of a buffer containing 20 mM ammonium bicarbonate pH 8.0, 1 % SDS, 100 mM beta-mercaptoethanol and incubated for 30 min at 65 °C. The tube was then placed in a magnet for 2 min. The supernatant was collected to which 20 μL of 0.25 M KCl was added to eliminate SDS by precipitation. To lower KCl concentration the supernatant (40 μL) was collected, diluted 10X with 20 mM ammonium bicarbonate pH 8.0 and concentrated by using a Microcon YM-3 centrifugal filter (Millipore). The concentrated sample was treated with PNGase F (2.5 units) in a final volume of 20 μL for 3 h at 37 °C. Gels were stained either with QC Colloidal Coomassie Stain (Bio-Rad) or Silver (BioRad Silver Stain Plus Kit) following manufacturer’s instructions.

### Western blot analysis

Western blot analysis was done essentially as described [2] using pull-down proteins extracted from 2 × 10^7^ 5g12e8-coupled Dynabeads. Serial Western blot analyses of proteins pulled down—hDEspR, hGalectin-1, hRab-1b and hTMED10—were done sequentially by stripping and re-probing the same blot in the following order: 1st, human-specific DEspR mAb (5g12e8 40 μg/ml, secondary anti-mouse IgG at 1:20,000), 2nd, Rab-1b polyclonal antibody (pAb) (Santa Cruz Biotechnology, cat # sc-599, at 1 μg/ml, secondary anti-rabbit IgG at 1:20,000), 3rd, human-specific Galectin-1 (Abcam, cat # ab25138 at 0.4 μg/ml, secondary anti-rabbit IgG 1:20,000) and 4th, pAb TMED10 (Abcam, cat # ab72666, at 10 μg/ml, secondary anti-rabbit IgG 1:20,000).

Serial Western blot analyses of U87 tumor cell membrane and Cos1-hDEspR cell membrane pull-downs were done sequentially by stripping and re-probing the same blot in order: 1st, hDEspR mAb (5g12e8 40 μg/ml, secondary anti-mouse IgG at 1:20,000) and then 2nd, hGalectin-1 (Abcam, cat#ab25138, at 0.4 μg/ml, secondary anti-rabbit IgG 1:20,000).

Serial western blot of U87 tumor membrane pull-down was also reacted sequentially by stripping and re-probing the same blot using first the following in order: 1st, hDEspR mAb (6g8g7 40 μg/ml, secondary anti-mouse IgG at 1:20,000), and 2nd, hDEspR mAb (5g12e8 40 ug/ml, secondary anti-mouse IgG at 1:20,000).

Immunoreactive proteins were detected by chemi-luminescence using the ECL Western Detection kit (GE Healthcare) as per manufacturer’s specifications.

### Mass spectrometry analysis

Mass spectrometry of pull-down proteins was custom-performed by Creative Proteomics (Shirley, NY). Briefly, proteins were reduced for 40 min with 5 mM dithiothreitol in 25 mM NH4HCO3 at room temperature and alkylated for 40 min with 15 mM iodoacetamide in 25 mM NH4HCO3 in the dark. After washed and dehydrated, the alkylated samples were digested overnight at 37 °C with trypsin in a 1:50 enzyme-to-substrate ratio (Promega, V5113). Following digestion, the peptide mixtures were acidified with trifluoroacetic (TFA) to 1 %, and desalted by home-made C18 tips. Finally, the desalted peptide samples were dried and dissolved in 10 μL of 0.1 % formic acid in water and subjected to nanoLC-MS/MS analysis in a Q Exactive mass spectrometer. The raw MS files were analyzed and searched against Uniprot human protein sequence database using Proteome Discoverer 1.4 (Thermo Fisher Scientific, USA). The parameters were set as follows: the protein modifications were carbamidomethylation (C) (fixed), oxidation (M) (variable), and myristyl on glycine (variable), Asn to Asp (variable); the enzyme specificity was set to trypsin; the maximum missed cleavages were set to 2; the precursor ion mass tolerance was set to 10 ppm, and MS/MS tolerance was 0.6 Da.

### Immunofluorescence analysis of subQ xenograft U87-CSC tumors

Double immunofluorescence staining was done as described [3]. Human-specific anti-DEspR mAb (5g12e8) and human-specific anti-Galectin-1 antibody (Abcam, cat#ab25138) were labeled with AlexaFluor(AF)-488 or AF568, and used at 1 μg/ml for anti-Galectin-1 and at 100 μg/ml for 5g12e8 mAbs on fixed, paraffin-embedded sections following antigen-retrieval. Digital photomicroscopy was done using a Zeiss Axioskop fluorescence microscope with auto-exposure settings.

### FACS analysis of U87-CSCs, MB-231-CSCs, H460-CSCs and Panc1-CSCs

U87-CSCs, MB-231-CSCs, H460-CSCs and Panc1-CSCs were incubated in ice-cold Hank’s balanced salt solution (HBSS, Invitrogen, NY) plus 2 % FBS containing: (a) 10 μg/ml AF-568 labeled 7c5b2 mAb, or (b) 10 μg/ml AF-568 labeled IgG2b as isotype control. Duplicate samples were incubated for 20 min at 4 °C, washed, resuspended in 1 % FBS/HBSS, 1 % PFA, filtered and analyzed on an LSR-II-FACS instrument. Analysis was done using FloJo Flow Cytometry Analysis Software (http://www.FloJo.com).

### DEspR-inhibition of CSC-growth

DEspR-inhibition studies were performed as described [3]. CSCs (2000/well) were seeded in ultra-low attachment 96-well plate and treated with different blocking anti-hDEspR mAbs (5g12e8, 7c5b2 and 6g8g7) at 100 μg/ml, compared with control non-treated CSCs with six replicates for each. CSCs were cultured in optimal (5 % CO2, humidified incubator at 37 °C) non-adherent conditions. Anti-hDEspR (5g12e8, 7c5b2 and 6g8g7) mAbs were added at seeding, day-2 and day-4. Live and dead CSCs were counted using Trypan Blue on day-5.

### RNA sequence analysis

The NCBI Sequence Read Archive was searched on 7/17/2013 with a query sequence provided by the Genbank accession for “Homo sapiens dual endothelin-1(VEGFsp)/angiotensin II receptor (DEAR) mRNA, complete cds,” gi|144,954,325|gb|EF212178.1|, against 727 sequencing runs (Additional file 5).

### Live cell imaging

Fluorescence and transmitted light images were acquired with a Zeiss LSM 710 Duo confocal microscope (Carl Zeiss, Thornwood NY). Excitation was from a 561 nm DPSS laser. A 63X 1.4 NA planapochromat oil immersion objective was used. The cells were in 35 mm dishes with coverglass bottoms. Emission was collected from 575–725 nm. Cells were maintained under physiological conditions using a Pecon stage-top incubation system which maintained 37° centigrade and 5 % CO2. Analysis was done using Image J (Image J, Wayne Rasband, NIH) with identical settings for brightness and contrast adjustments for all images. Live cell imaging with AF-568 labeled isotype IgG2b control was performed in identical conditions. At 60 min, cells were fixed briefly and mounted with Vectashield mounting medium with DAPI (Vector Laboratories, CA, Cat.# H-1200). Photomicroscopy was done using a Zeiss Axioskop fluorescence microscope (Carl Zeiss, Thornwood NY) with differential interference contrast (DIC).

### Statistical analysis

All data were analyzed for normality and descriptive statistics. The following statistical tests were performed using SigmaPlot 11.0 or PRISM 5: one-way analysis of variance (ANOVA) followed by Tukey multiple comparisons test (MCT) for CSC-growth inhibition experiments. A *P* < 0.05 was considered statistically significant.

## Additional files

10.1186/s12867-016-0066-8 NCBI designation of Dear (alias DEspR) gene as pseudogene—updated May 2016.
10.1186/s12867-016-0066-8 Sanger dideoxy-sequencing of DEspR spanning amino acid 14 (aa14) codon-TGG position within a Yamakawa compression-motif [Y-G-N1, 2-A-R]: DEspR 5′ T-G-G-A-A, shows two G’s (GG), and downward slippage/compression of the two G’s towards the 5′ T. A 3-nt long stem-loop structure spans the compression motif region in human DEspR but not in rat DEspR.
10.1186/s12867-016-0066-8 Unedited RNA-Seq entries showing the questioned two G’s with insertion A or TA document difficult sequencing region as seen in manual sequencing.
10.1186/s12867-016-0066-8 Protein profile pulled-down (Creative Proteomics). Proteins pulled-down with anti-DEspR mAb 5g12e8 from U87 CSC membranes.
10.1186/s12867-016-0066-8 RNA-seq analysis. List of 727 sequencing runs searched with a DEspR query sequence in the NCBI Sequence Read Archive.

## Abbreviations

DEspR: dual endotheiln-1/VEGFsp receptor
VEGFsp: vascular endothelial growth factor signal peptide
CSC: cancer stem-like cell
ARMS: allele-specific amplification-refractory mutation system
ORF: open reading frame
FBXW7: F-box and WD repeat domain containing 7
mAb: monoclonal antibody
Gal-1: galectin-1
Gal-3: galectin-3
TMED10: transmembrane emp24 domain-containing protein 10
Rab-1b: ras-related protein Rab-1B
PNGase F: peptide-N-glycosidase F
MS: mass spectrometry
