# Supplementary material for: Confirmation of translatability and functionality certifies the dual endothelin1/VEGFsp receptor (DEspR) protein
Source: BMC Mol Biol. 2016 Jun 14;17:15. doi: 10.1186/s12867-016-0066-8 (PMC4906906; doi:10.1186/s12867-016-0066-8)
Supplement: Supplementary file 1 — 10.1186/s12867-016-0066-8 NCBI designation of Dear (alias DEspR) gene as pseudogene—updated May 2016. [file 12867_2016_66_MOESM1_ESM.pdf]

Figure S1. NCBI Designation of DEspR (*Dear)* as pseudogene

http://www.ncbi.nlm.nih.gov/gene/102191832[5/16/2016 4:05:57 PM]

DEAR dual endothelin-1(VEGFsp)/angiotensin II receptor pseudogene [*Homo sapiens* (human)]

Gene ID: 102191832, updated on 7-May-2016

**Gene symbol**	DEAR
**Gene description**	dual endothelin-1(VEGFsp)/angiotensin II receptor pseudogene
**See related**	Ensembl:ENSG00000270751
**Gene type**	pseudo
**RefSeq status**	VALIDATED
**Organism**	Homo sapiens
**Lineage**	Eukaryota; Metazoa; Chordata; Craniata; Vertebrata; Euteleostomi; Mammalia; Eutheria; Euarchontoglires; Primates; Haplorrhini; Catarrhini; Hominidae; Homo
**Also known as**	DEspR
**Annotation information**	Note: The human DEAR gene is represented as a transcribed pseudogene based on abundance of available evidence. There is a conflicting report about this gene being protein-coding (PMID:17446437). [04 Nov 2013]
