# Supplementary material for: Confirmation of translatability and functionality certifies the dual endothelin1/VEGFsp receptor (DEspR) protein
Source: BMC Mol Biol. 2016 Jun 14;17:15. doi: 10.1186/s12867-016-0066-8 (PMC4906906; doi:10.1186/s12867-016-0066-8)
Supplement: Supplementary file 2 — 10.1186/s12867-016-0066-8 Sanger dideoxy-sequencing of DEspR spanning amino acid 14 (aa14) codon-TGG position within a Yamakawa compression-motif [Y-G-N1, 2-A-R]: DEspR 5′ T-G-G-A-A, shows two G’s (GG), and downward slippage/compression of the two G’s towards the 5′ T. A 3-nt long stem-loop structure spans the compression motif region in human DEspR but not in rat DEspR. [file 12867_2016_66_MOESM2_ESM.docx]

**Figure S2.** Sanger dideoxy-sequencing of DEspR spanning amino acid 14 (aa14) codon-TGG position within a Yamakawa compression-motif [Y-G-N_1, 2_-A-R]: DEspR 5’ T-G-G-A-A, shows two G’s (GG), and downward slippage/compression of the two G’s towards the 5’ T. A 3-nt stem-loop that is present in human-DEspR (hDEspR) but not in rat-DEspR (rDEspR) can also be expected to contribute to the difficult sequencing region.

5 pmol 25 pmol 100 pmol

G A T C G A T C G A T C


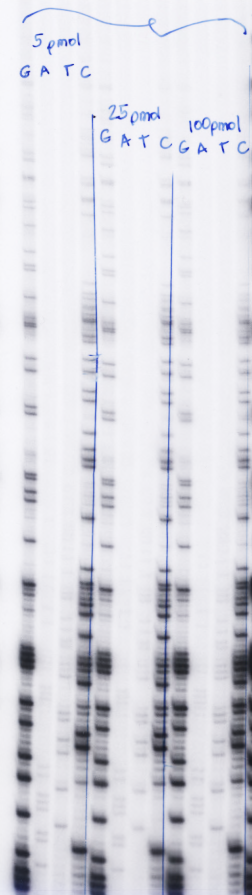


5’

3’

**G**

**G**

**G**

**G**

**G**

**G**

**G**

**G**

**C**

**G**

**G**

**T**

**T**

**G**

**C**

**A**

**A**

**T**

G

G

G

G

**T**

**T**

**T**

**T**

**G**

**G**

**G**

**G**

**C**

**C**

**T**

**T**

**A**

**A**

**A**

**A**

**G**

**C**

**G**

**C**

**Compression site**

Motif ^5’^ Y G N_1-2_ A R ^3’^

DEspR ^5’^ T G G A A ^3’^

Y: pyrimidine; R: purine


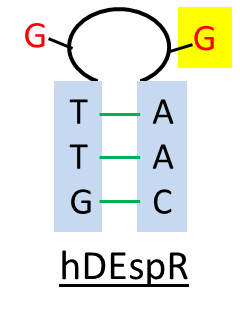

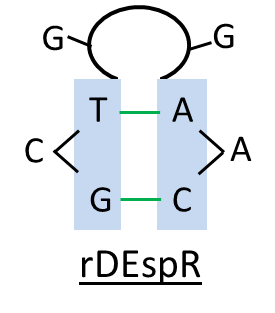


Manual sequencing performed using DNA Sequenase v.2; αP^32^ dATP; dGTP; 3 primer conditions: 5-, 25-, 100-pmol; and 6% sequencing gel run for 6-hours at constant voltage shows two G’s (GG) comprising the T-G-G codon for tryptophan (W). The two G’s are within a Yamakawa compression motif in the human DEspR sequence: 5’ T-G-G-A-A 3’, and are compressed 5’, ie, downwards towards a T in the sequencing gel-run, but leave a G band-shadow stronger than the A-band. The A-bands are faint and fainter than G-bands. Additionally, a 3-nt stem-loop structure spans the difficult sequencing segment of the two G’s (GG) in humans but not in rat-DEspR (rDEspR). The TGG codon is not questioned in rat sequence.

In the difficult sequencing region prone to compression per Yamakawa compression-motif (Yamakawa et al 1996), presence of two G’s is supported by presence of parallel two C’s across. In the difficult sequencing region, every known, uncontested-G has a parallel C. In contrast, known, uncontested C’s do not have a parallel G. Based on research standards, the automated sequencing-generated “A” is the sequencing error, not the Sanger sequencing we obtained detecting the “G”at said nt 109 (or aa#14 position), since Sanger sequencing is the final determinant of sequence discrepancy (Brodin et al 2013) – especially since systematic sequencing errors occur (Meacham et al 2011). As published by Brodin et al 2013, “any difference from the Sanger sequence is defined as a sequencing error.”

References:

Yamakawa H, Nakajima D, Ohara O. 1996. Identification of sequence motifs causing band compressions on human cDNA sequencing. DNA Res 3:81-6.

Brodin J, Mild M, Hedskog C, Sherwood E, Leitner T, Andersson B, Albert J. 2013. PCR-induced transitions are the major source of error in cleaned up ultra-deep pyrosequencing data. PlosOne 8(7) e70388.

Meacham F, Boffelli D, Dhahbi J, Martin DIK, Singer M, Pachter L. 2011. Identification and correction of systematic error in high-throughput sequence data. BMC Bioinformatics 12:451.
