# Supplementary material for: Confirmation of translatability and functionality certifies the dual endothelin1/VEGFsp receptor (DEspR) protein
Source: BMC Mol Biol. 2016 Jun 14;17:15. doi: 10.1186/s12867-016-0066-8 (PMC4906906; doi:10.1186/s12867-016-0066-8)
Supplement: Supplementary file 3 — 10.1186/s12867-016-0066-8 Unedited RNA-Seq entries showing the questioned two G’s with insertion A or TA document difficult sequencing region as seen in manual sequencing. [file 12867_2016_66_MOESM3_ESM.pdf]

Figure S3. Some RNA-Seq sequences show two G's with extra "A" or "TA"

1) HEK46AV01A5IHP. Sequence ID: **gnl|SRA|ERR169824.68765.1** Length: 895 Number of Matches: 1
Range 1: 409 to 699 [truncated sbj ct 580 – 699]

Score	Expect	Identities	Gaps	Strand	Frame
525 bits(284)	5e-145()	289/291(99%)	2/291(0%)	Plus/Minus	

```
Query  82   AGGGAGC-AATGAAATGAAGTCTCGTTGG-AACTGGGGTTCTATCACTTGCATCATATGT  139
            ||||||| ||||||||||||||||||||| ||||||||||||||||||||||||||||||
Sbjct  699  AGGGAGCAAATGAAATGAAGTCTCGTTGGAAACTGGGGTTCTATCACTTGCATCATATGT  640

Query  140  TTTACTTGTGTTGGTTCACAACTATCAATGAGTTCATCTAAAGCAAGCAATTTCTCTGGT  199
            ||||||||||||||||||||||||||||||||||||||||||||||||||||||||||||
Sbjct  639  TTTACTTGTGTTGGTTCACAACTATCAATGAGTTCATCTAAAGCAAGCAATTTCTCTGGT  580
```

2) E56FR8H01C88XQ. Sequence ID: **gnl|SRA|SRR013993.103029.2** Length: 226 Number of Matches: 1
Range 1: 74 to 212 [truncated 93-212]

Score	Expect	Identities	Gaps	Strand	Frame
195 bits(105)	2e-45()	129/139(93%)	8/139(5%)	Plus/Minus	

```
Query  82   AGGGAGC-AATGAAATGAAGTCTCGTTGG--AACTGGGGTTC-TATCACTTGCATCATAT  137
            ||| ||| |||||||||||||||||||||  ||||||||||| |||||||||||||||||
Sbjct  212  AGGTAGCGAATGAAATGAAGTCTCGTTGGTAAACTGGGGTTCGTATCACTTGCATCATAT  153

Query  138  GTTTTACTTGT-GTTGG-TTC-ACAACTATCAATGAGTTCATCTAAAGCAAGCAATTTCT  194
            ||||||||||| ||||| ||  ||||||||||||||||||||||||||||||||||||||
Sbjct  152  GTTTTACTTGTTGTTGGGTTTNACAACTATCAATGAGTTCATCTAAAGCAAGCAATTTCT  93
```

3) GAN14SU02ECA9M. Sequence ID: **gnl|SRA|SRR644608.4046991.2** Length: 496 Number of Matches: 1
Range 1: 391 to 482

Score	Expect	Identities	Gaps	Strand	Frame
134 bits(72)	4e-27()	87/93(94%)	6/93(6%)	Plus/Plus	

```
Query  92   GAAATGAAGT-C--TCGTTGG-AACTGGGGTTCTATCACTTGCATCATATGTTTTACTTG  147
            |||||||||| |  ||||||| ||||||||||||||||||||||||||||||||||||||
Sbjct  391  GAAATGAAGTCCGGTCGTTGGAAACTGGGGTTCTATCACTTGCATCATATGTTTTACTTG  450

Query  148  TGTTGGTTCACAACTATCAATGAGTTCA-TCTA  179
            |||||||||||| ||||||||||||||| ||||
Sbjct  451  TGTTGGTTCACA-CTATCAATGAGTTCACTCTA  482
```

4) GAN14SU06HEXAL. Sequence ID: **gnl|SRA|SRR644608.4390352.2** Length: 493 Number of Matches: 1
Range 1: 325 to 456 [truncated 340-456]

Score	Expect	Identities	Gaps	Strand	Frame
193 bits(104)	6e-45()	126/135(93%)	7/135(5%)	Plus/Minus	

```
Query  82   AGGGAG-CAATGAAATGAAGTCTCGTTGG-AACT-GGGGTTCTATCACTTGC-ATCATAT  137
            |||||| ||||||||||||||||| |||| |||| ||||||||||||||||| |||||||
Sbjct  456  AGGGAGACAATGAAATGAAGTCTC-TTGGAAACTCGGGGTTCTATCACTTGCTATCATAT  398

Query  138  GTTTTACTTGTGTTGGTTCACAACTATCAATGAGTTCATCTAAAGCAAGCAATTTCTCTG  197
              |||||||||||||| |||| ||||||||||||||||||||||||||||||||||||||
Sbjct  397  CGTTTACTTGTGTTGG-TCAC-ACTATCAATGAGTTCATCTAAAGCAAGCAATTTCTCTG  340
```

5) ERX145715 [From: Expt 2]. Sequence ID: **gnl|SRA|ERR169825.197680.1** Length: 886 Number of Matches: 1
Range 1: 498 to 813 [truncated 694-813]

Score	Expect	Identities	Gaps	Strand	Frame
326 bits(176)	5e-88()	276/317(87%)	38/317(11%)	Plus/Minus	

```
Query  91   TGAAA-TGAA-G-TCTCG-TTGG--AA-CTGGGG-TTC---TA-TCAC--TTG-CA-TCA  134
            ||||| |||| | ||||| ||||  || |||||| |||   || ||||  ||| || |||
Sbjct  813  TGAAATTGAAGGTTCTCGTTTGGTAAACCTGGGGTTTCGTTTATTCACTTTTGTCATTCA  754

Query  135  TATG--TTTTAC-TTG-TGTT--GG-TTC-ACAACT-ATCAATGAG-TTCATCTAAAG-C  183
            ||||  |||||| ||| ||||  || ||| |||||| ||||||||| ||||||||||| |
Sbjct  753  TATGTTTTTTACTTTGTTGTTAGGGTTTCTACAACTAATCAATGAGTTTCATCTAAAGAC  694
```
