# Supplementary material for: Confirmation of translatability and functionality certifies the dual endothelin1/VEGFsp receptor (DEspR) protein
Source: BMC Mol Biol. 2016 Jun 14;17:15. doi: 10.1186/s12867-016-0066-8 (PMC4906906; doi:10.1186/s12867-016-0066-8)
Supplement: Supplementary file 5 — 10.1186/s12867-016-0066-8 RNA-seq analysis. List of 727 sequencing runs searched with a DEspR query sequence in the NCBI Sequence Read Archive. [file 12867_2016_66_MOESM5_ESM.pdf]

RNA-Seq analysis.

The NCBI Sequence Read Archive was searched on 7/17/2013 with a query sequence provided by the Genbank accession for "Homo sapiens dual endothelin-1(VEGFsp)/angiotensin II receptor (DEAR) mRNA, complete cds," gi|144954325|gb|EF212178.1|, against 727 sequencing runs listed below.

SRR801066, SRR801053, SRR801050, SRR801060, SRR801031, SRR800863, SRR800943, SRR800910, SRR800890, SRR800887, SRR801027, SRR800903, SRR800951, SRR800877, SRR800915, SRR801033, SRR800889, SRR800917, SRR800970, SRR801045, SRR800867, SRR800885, SRR800881, SRR800944, SRR409083, SRR801014, SRR800873, SRR800870, SRR800940, SRR801026, SRR800864, SRR801042, SRR800982, SRR409084, SRR800998, SRR800996, SRR800980, SRR800896, SRR800984, SRR800880, SRR801016, SRR800960, SRR409002, SRR408996, SRR172884, SRR029585, SRR006766, SRR409098, SRR574617, SRR006762, SRR574627, SRR574612, SRR409109, SRR574630, SRR574624, SRR574632, SRR409088, SRR574614, SRR408992, SRR409122, SRR409106, SRR065397, SRR018370, DRR001735, SRR518131, SRR409072, SRR013994, SRR064489, SRR018365, SRR182394, SRR014004, SRR006765, SRR182366, SRR409082, SRR013997, SRR013986, SRR409150, SRR620226, SRR408997, SRR409077, SRR376912, SRR133576, SRR409148, SRR342054, SRR182376, SRR621474, SRR182372, SRR182383, SRR182388, ERR169827, SRR515314, SRR182371, SRR747232, SRR063335, SRR063343, SRR063347, SRR063346, SRR063348, SRR063344, SRR070668, SRR747766, SRR133629, SRR436889, SRR133632, SRR133637, SRR133589, SRR133634, SRR133617, DRR001757, SRR051920, DRR001753, SRR133594, DRR001755, SRR133591, SRR133609, SRR133613, SRR133635, SRR133573, SRR099374, SRR133623, SRR051923, SRR133615, SRR133586, SRR578234, DRR001730, DRR001759, SRR051914, SRR543533, SRR051917, DRR001736, SRR077239, SRR543506, SRR543502, SRR077247, SRR543503, SRR578258, SRR578239, SRR644784, SRR543519, SRR543518, SRR099317, SRR543528, SRR077253, SRR543525, SRR543522, SRR543512, SRR077250, SRR801067, SRR801064, SRR801065, SRR801055, SRR801059, SRR801039, SRR800971, SRR800945, SRR801015, SRR800955, SRR800919, SRR800931, SRR800999, SRR801029, SRR800987, SRR801023, SRR800967, SRR800859, SRR800941, SRR801009, SRR800927, SRR800909, SRR800893, SRR800973, SRR800981, SRR800882, SRR801021, SRR800985, SRR800994, SRR800906, SRR800925, SRR800898, SRR801049, SRR800913, SRR065400, SRR800876, SRR800904, SRR800886, SRR800918, SRR409104, SRR801034, SRR801032, SRR801044, SRR800920, SRR800966, SRR800884, SRR065402, SRR800948, SRR027922, SRR800952, SRR800956, SRR065401, SRR800866, SRR800912, SRR800964, SRR409121, SRR124144, SRR029581, SRR574628, SRR029582, SRR099206, SRR018356, SRR574626, SRR018354, SRR574629, SRR574622, SRR409001, SRR409074, SRR018355, SRR409114, SRR409078, SRR013990, SRR409112, SRR051919, SRR018360, SRR182392, SRR018368, SRR409111, SRR182395, ERR169820, SRR013987, SRR013998, SRR013996, SRR013992, SRR443375, SRR620224, SRR620225, SRR620222, SRR182369, SRR621466, SRR409149, ERR169823, SRR133627, SRR747758, SRR133571, ERR169824, SRR133601, SRR063334, SRR063336, SRR063342, SRR063340, SRR830956, SRR133574, SRR182382, SRR400159, ERR169828, SRR847411, SRR133604, SRR747761, SRR520409, SRR654170, SRR747767, SRR099373, SRR133633, SRR578242, SRR654172, SRR133612, SRR847343, SRR847342, SRR133608, SRR051922, SRR578261, SRR133638, SRR568013, SRR133590, SRR578248, DRR001731, SRR578238, SRR543521, SRR578236, DRR001733, DRR001742, DRR001743, SRR578251, SRR543520, SRR543505, SRR543508, SRR543515, SRR644608, SRR578254, SRR543516,

SRR077251, SRR077252, SRR077248, SRR578260, SRR574618, SRR801057, SRR801062, SRR801058, SRR065195, SRR801047, SRR800907, SRR801011, SRR801007, SRR800883, SRR801041, SRR801035, SRR800879, SRR800875, SRR065198, SRR800969, SRR800923, SRR801001, SRR800953, SRR800933, SRR800905, SRR800959, SRR800869, SRR801030, SRR800897, SRR801006, SRR800993, SRR800902, SRR801002, SRR800860, SRR800968, SRR065194, SRR800916, SRR065393, SRR801022, SRR800936, SRR800932, SRR800972, SRR800908, SRR801008, SRR800868, SRR409105, SRR801004, SRR800924, SRR409089, SRR800992, SRR494404, SRR408999, SRR099551, SRR409125, SRR029584, SRR408994, SRR574623, SRR018352, SRR409097, SRR574625, SRR574631, SRR064504, SRR065398, SRR574611, SRR409113, SRR409000, SRR019142, SRR182397, SRR051918, SRR013985, SRR014000, SRR518130, SRR064474, SRR018361, SRR018367, SRR014003, SRR018359, SRR013995, SRR409080, SRR182398, SRR064505, SRR409101, SRR013991, SRR182390, SRR133575, SRR620223, SRR064491, SRR182375, SRR182368, SRR621472, SRR621476, SRR621470, SRR621469, SRR621475, SRR133596, SRR182365, SRR182381, SRR182378, SRR770500, SRR063341, SRR063339, SRR063338, SRR051911, SRR654171, SRR182362, SRR133630, SRR051912, SRR133597, SRR182386, SRR133636, SRR133620, SRR133572, SRR133621, SRR133602, SRR133603, SRR133584, SRR133624, SRR133587, SRR182385, SRR133605, SRR099371, SRR133626, SRR133616, DRR001752, SRR182379, SRR133614, SRR133607, DRR001756, SRR051924, DRR001750, SRR133625, SRR578241, DRR001744, DRR001734, DRR001740, SRR578233, DRR001745, SRR578257, SRR578235, SRR077246, SRR578247, SRR543507, SRR099376, SRR578243, SRR543517, SRR578253, SRR543523, SRR543524, SRR578259, SRR543511, SRR801052, SRR800911, SRR800935, SRR800939, SRR801003, SRR800899, SRR800991, SRR065399, SRR800947, SRR801043, SRR800895, SRR800861, SRR800983, SRR801019, SRR800921, SRR800957, SRR800901, SRR801046, SRR800989, SRR800977, SRR801013, SRR801040, SRR800894, SRR801017, SRR801010, SRR801025, SRR800974, SRR800930, SRR800954, SRR800961, SRR800914, SRR800878, SRR065394, SRR800922, SRR800965, SRR800858, SRR800978, SRR800986, SRR801018, SRR800926, SRR800976, SRR801036, SRR801012, SRR801048, SRR801024, SRR172885, SRR494405, SRR409155, SRR574616, SRR029578, SRR735691, SRR018357, SRR409116, SRR018369, SRR574615, SRR574619, SRR006764, SRR409099, SRR409107, SRR409151, SRR409108, SRR409103, SRR065395, SRR408998, DRR001741, SRR018371, SRR409117, SRR182396, SRR013993, SRR018363, SRR018358, SRR013999, SRR018364, SRR013989, SRR409110, SRR182389, SRR071999, SRR409003, SRR409124, SRR182391, SRR409079, SRR494402, SRR133577, SRR409087, SRR072001, SRR620221, SRR014001, SRR072000, SRR064490, SRR747764, SRR515311, SRR182377, SRR515312, SRR621471, SRR520407, SRR515313, SRR182373, ERR169825, SRR182380, SRR063349, SRR133583, SRR747768, SRR520408, SRR133606, SRR436888, SRR747763, SRR747760, SRR133579, SRR133631, SRR133598, SRR051921, DRR001748, SRR133600, SRR099319, SRR133582, SRR133622, DRR001749, SRR133595, SRR133610, SRR133611, SRR133639, SRR578244, DRR001751, SRR543532, DRR001746, DRR001732, DRR001747, SRR051913, DRR001760, DRR001737, DRR001738, SRR578256, SRR578262, SRR543504, SRR578255, SRR543514, SRR543531, SRR543529, SRR543509, SRR543530, SRR077244, SRR077242, SRR077249, SRR543527, SRR077245, SRR801054, SRR801051, SRR801056, SRR801063, SRR801061, SRR800891, SRR065391, SRR800963, SRR800995, SRR800871, SRR800975, SRR801038, SRR800865, SRR800979, SRR800937, SRR800997, SRR800862, SRR800949, SRR800942, SRR800962, SRR801037, SRR800934, SRR800929, SRR800888, SRR801005, SRR065392, SRR800938, SRR065193, SRR800950, SRR800946, SRR800874, SRR800990, SRR801028, SRR801000, SRR800892, SRR409100, SRR800900, SRR800928, SRR800988, SRR800958, SRR027923,

SRR801020, SRR800872, SRR409102, SRR408995, SRR409081, SRR494403, SRR409123, SRR029583, SRR574610, SRR018351, SRR018353, SRR574620, SRR574633, SRR574613, SRR574621, SRR409120, SRR182367, SRR409134, SRR409073, SRR006763, SRR065396, SRR064488, SRR018366, SRR018374, SRR013988, SRR018362, SRR018373, SRR409115, SRR018372, SRR013983, SRR409004, SRR013984, ERR169819, SRR409075, SRR006767, SRR182393, SRR014002, SRR409156, SRR408993, ERR169821, ERR169818, SRR620220, SRR409076, SRR765688, SRR620227, SRR064492, SRR065199, SRR568014, SRR654169, SRR747759, SRR621473, SRR621477, SRR621468, SRR747256, SRR409147, SRR182387, SRR182370, SRR133585, SRR182363, SRR182374, SRR400158, SRR063337, SRR568012, SRR051910, SRR063345, ERR169826, SRR133628, SRR747785, SRR182364, SRR099372, SRR133580, SRR099375, SRR133581, SRR133599, SRR133592, DRR001754, SRR133593, DRR001758, SRR847391, SRR133578, SRR133618, SRR133619, SRR133588, SRR182384, SRR051916, SRR051915, SRR578240, DRR002075, DRR001739, SRR578237, DRR001761, SRR578249, SRR578252, SRR099318, SRR077243, SRR578250, SRR543510, SRR077240, SRR543526, SRR543513, SRR578246, SRR077241, SRR578245.
